# Profiling of Mycoplasma gallisepticum Ribosomes

**Published:** 2015

**Authors:** G. Y. Fisunov, D. V. Evsyutina, A. A. Arzamasov, I. O. Butenko, V. M. Govorun

**Affiliations:** Scientific research institute of physical-chemical medicine, Malaya Pirogovskaya, 1a, 119435, Moscow, Russia

**Keywords:** mycoplasma, ribosome, ribosome profiling

## Abstract

The development of high-throughput technologies is increasingly resulting in identification of numerous cases of low correlation between mRNA and the protein level in cells. These controversial observations were made on various bacteria, such as *E. coli*, *Desulfovibrio vulgaris*, and *Lactococcus lactis. *Thus, it is important to develop technologies, including high-throughput techniques, aimed at studying gene expression regulation at the level of translation. In the current study, we performed proteomic profiling of *M. gallisepticum *ribosomes and identified high abundant noncanonical proteins. We found that binding of mRNAs to ribosomes is mainly determined by two parameters: (1) abundance of mRNA itself and (2) complimentary interactions between the 3’ end of 16S rRNA and the ribosome binding site in the 5’-untranslated region of mRNA.

## INTRODUCTION

System research using the so-called “omics technologies” increasingly reveal unexpected phenomena and new regulated events that simply cannot be determined using either an omics technology alone or traditional methods of analysis. However, the joint analysis of data obtained from the quantitative measurement of RNA or protein and peptide levels inevitably creates a significant number of artifacts due to mistakes in each of the methods used. This fact requires careful cross analysis and additional confirmation of the obtained data using alternative methods or orthogonal processing. For this reason, prokaryotes are used as subjects for testing a common methodology for the analysis of the joint behavior of macromolecules in living systems and their reciprocal impact.

The European Molecular Biology Laboratory has established a project dedicated to studying the causative agent of human respiratory diseases, a representative of the class *Mollicutes *– *Mycoplasma pneumoniae *[1, 2]. Then, our group selected another representative of this class (*M. gallisepticum*) to carry out a similar study. Soon afterwards, American researchers developed a computer model of the metabolism and adaptive responses of the smallest self-replicating bacteria – *M. genitalium *[3]. Despite these successes, a large number of issues remains unexplored, requiring additional methods for accessing dynamic processes during expression of the minimum set of genetic information encoded in the genome of *Mycoplasma*.

*Mollicutes*, which include *M. gallisepticum, *are characterized by a significant genome reduction. The average size of the genome of *Mycoplasma *typically ranges from 800 thousand to 1 million bp (1 million in *M. gallisepticum*) [4]. Due to genome reduction, *Mycoplasma* lost the well-known mechanisms of regulation of gene expression [5].

As we have showed earlier, *M. gallisepticum *responds to stress at the transcriptional level [6]. At the same time, these changes are generally slightly reflected at the level of translation [6]. This phenomenon may be caused by two reasons: (1) the rate of translation in *M. gallisepticum *is not enough to reveal changes in the protein level during the experiment (30 min), (2) mRNA binds selectively to ribosomes during the stress response. The mechanism of selective attaching of mRNA to ribosomes can be realized through interaction with antisense RNA that blocks the ribosome binding site [7]. Moreover, even within the same cell, ribosomes may differ from each other both in the nucleotide sequence of rRNA [8] and in protein composition. For example, ribosomes in *Escherichia coli, *which do not include S1 protein, translate mainly leaderless transcripts [9]. Ribosomes may bind regulatory proteins modulating translation in particular transcripts [10]. Ribosomes may bind noncanonical proteins whose primary function is not related to translation. For example, glycogen synthase in *Sascharomyces cerevisiae *can affect translation of various RNAs [11].

The development of high-throughput technologies has led to the accumulation of a large database on transcription and translation in the entire cell. According to the classical view, protein level is generally determined by the level of the corresponding mRNA; however, in some cases, it is not. High-throughput technologies have increased the number of cases when the protein level does not correlate with the level of mRNA. Such data were obtained for a variety of bacteria, including* E. coli *[12], *Desulfovibrio vulgaris *[13], and *Lactococcus lactis *[14]. The Pearson correlation coefficient between the level of mRNA and protein level, according to the published data, can vary from 0.53 to 0.19, depending on the type and state of the bacteria. Significant progress in studying the regulation of gene expression at the translational level was achieved with a technology of ribosome profiling [15] which enables observing translation almost in real time. Thus, the stage of binding mRNA to the ribosome and the process of translation is an extremely significant part in the regulation of gene expression in bacteria. Since *Mollicutes *in general and *M. gallisepticum *in particular are characterized by the lack of transcriptional regulatory mechanisms, regulation of gene expression at the translational level can be perhaps the most significant part in determining protein abundance in the cell.

In the present study, a high-throughput proteomic profiling of *M. gallisepticum *ribosomes to determine the composition of ribosomes and transcriptional profiling of ribosome-bound mRNA using real-time PCR were performed.

## EXPERIMENTAL SECTION

**M. gallisepticum S6 culturing**

*M. gallisepticum *S6 was cultured in a liquid medium (20 g/l Tryptose, 5 g/l NaCl, 1.3 g/l KCl, 3 g/l Tris, 5% yeast dialysate, 6% horse serum, and 1% glucose; pH 7.4) to mid-logarithm growth phase as described in [16].

**Ribosome purification**

Chloramphenicol was added to 12 ml of *M. gallisepticum* cell culture to a final concentration of the solution of 100 μg/ml, which was thoroughly mixed and incubated for 5 min on ice. Then, the cells were pelleted by centrifugation at 4,500 g for 20 min (4°C). The supernatant was removed, and the cell pellet, which was obtained from 50 ml of culture, was resuspended in 500 μl of lysis buffer containing 20 mM HEPES, 100 mM NaCl, 6 mM MgCl_2_, 2 mM spermidine, 100 μg/ml chloramphenicol, 5 μl protease inhibitor (GE Healthcare), and 200 units of RNase inhibitor (Thermo Scientific) (pH 7.5). Following the resuspension, 15 μl of NP-40 was added to the buffer and the composition was mixed thoroughly. Then, the cell lysate was frozen for at least 1 hour at –75°C. The cell lysate was purified by centrifugation at 20,000 g for 20 min (4°C). The supernatant was collected and fractionated by centrifugation on a sucrose step gradient.

Sucrose step gradient was created in a 5 ml polycarbonate tube by layering sucrose solutions of different densities using a pipette. The volume of each layer was 750 μl, and the difference in density was 10%. In this study, we used 10–50% sucrose gradients (a total of 5 layers). Sucrose solution was prepared using the same buffer as for cell lysis (without adding NP-40, chloramphenicol, and inhibitors of proteases and RNases). The mixture was centrifugated at 50,000 rpm (200,620 g on average) for 1 h at 4°C, using the Optima centrifuge (Beckman Coulter) and the MLS 50 swinging bucket rotor (Beckman Coulter). 200 μl aliquots of fractions were collected using a pipette.

**Table:** Spearman correlation between biological replicates of ribosome-bound mRNA sample. Levels of mRNAs were measured by real-time PCR

	Rep1	Rep2	Rep3
Rep1	1	0.87	0.92
Rep2	0.87	1	0.90
Rep3	0.92	0.90	1

**RNA isolation from fractions**

Each fraction was added to 400 μl of Trizol LS reagent (Life Technologies). The content was thoroughly mixed, and 200 μl of chloroform was added. The composition was then mixed again and centrifuged for 10 minutes at 16,000 g (4°C). The supernatant was collected and resuspended in an equal volume of isopropanol. The sample was incubated for at least 1 h at –20°C. RNA was pelleted by centrifugation at 16,000 g for 20 min (4°C). The pellet was washed with 80% (v/v) ethanol. The RNA sample was then dissolved in 10 μl of water (Panreac). RNA abundance in the fractions was measured by quantitative real-time PCR. The experiment was conducted in 3 biological replicates.

**Protein extraction from fractions and trypsin digestion**

For protein precipitation, each fraction was diluted 10-fold with deionized water and trichloroacetic acid (Sigma-Aldrich) was added to a final concentration of 10% (v/v). The mixture was left at 4°C overnight and then centrifugated for 15 min at 16,000 g. The pellet was washed twice with 1 ml of cold acetone (Pancreac) to remove residual trifluoroacetic acid.

Protein pellets were redissolved in 25–35 μl of 50 mM ammonium bicarbonate solution (Pancreac) containing 0.5% RapiGest SF (Waters) and 1 μl of Nuclease Mix (GE Healthcare). Then, the mixture was left for 30 min at 4°C, incubated for 5 min at 100°C, and centrifuged for 10 min at 16,000 g. The supernatant was collected, and the protein content was determined in each sample using bicinchoninic acid (Bicinchoninic Acid Protein Assay Kit, Sigma-Aldrich). In order to reduce disulfide bonds, dithiothreitol (Bio-Rad) was added to the protein solution to a final concentration of 10 mM (the reaction was conducted on a shaker (600 rpm) for 30 minutes at 60°C). The subsequent alkylation of cysteine residues by iodoacetamide (final concentration of 30 mM; Bio-Rad) was performed for 30 min at room temperature in the dark. Then, trypsin (Trypsin Gold, Mass Spectrometry Grade, Promega) was added to protein samples; trypsin:protein ratio (w/w) was 1:50. Trypsin digestion was performed during 16 hours at 37°C. The reaction was stopped by adding 10% trifluoroacetic acid (Sigma-Aldrich) (pH after trifluoroacetic acid addition should be 2.0). Then, the sample was incubated for 45 min at 37°C and centrifuged (15 min at 16,000 g) to remove RapiGest SF. The mixture of tryptic peptides was additionally purified by solid phase extraction using Discovery DSC-18 mini columns (Supelco) according to the manufacturer’s recommendations. For further mass spectrometry analysis, the eluate was dried in the CentriVap vacuum concentrator (Labconco) and dissolved in 10 μl of 3% acetonitrile solution containing 0.1% formic acid.

**RNA isolation from cell culture**

RNA was extracted from the cell culture according to [16]. Triple volume of Trizol LS reagent (Thermo Scientific) was added to aliquotes of cell culture. Phase separation was induced by adding chloroform (80 μl per 100 μl of cell culture). Samples were centrifuged at 10,000 g for 15 min (4°C). Then, the RNA samples were reprecipitated with isopropanol (1:1 v/v).

**cDNA synthesis and qPCR**

cDNA synthesis and real-time PCR were performed as described in [16]. RNA samples were treated with DNase I (Thermo Scientific). Then, cDNA was synthesized with reverse transcriptase (H minus Reverse Transcriptase, Thermo Scientific) and random hexamers. RiboLock RNase inhibitor (Thermo Scientific) was used to enhance the stability of RNA.

Quantitative real-time PCR (qRT-PCR) was performed on the C1000 Touch thermal cycler (Bio-Rad) with the CFX96 optical module (Bio-Rad). For PCR, 10X PCR buffer (Lytech) (1.5-fold final dilution), 10X dNTP mixture (Lytech), Taq-polymerase (Lytech), SYBR Green I dye (Life Technologies), 5 pmol primers, and 2% formamide were used. Data normalization was carried out according to the average mRNA abundance in 21 housekeeping genes (*eno, gaphd, tpiA, tuf, tsf, acoA, acoB, aceF, ldh, ackA, pgk, fba, pgi, pfkA, gpmI, pykF, tktA1, rpiB, eutD, prsA, *and *lpd*). The same as in [6] primers were used for qRT-PCR.

**Identification of Proteins**

Chromatography-mass spectrometry analysis of peptide extracts was performed using the Q-Exactive HF mass spectrometer (Thermo Fisher Scientific) coupled with the Ultimate 3000 RSLCnano LC system (Dionex) through the Nanospray Flex ion source (Thermo Fisher Scientific).

Peptides were separated by inversed-phase chromatography using Acclaim PepMap precolumn (C18 stationary phase, length of 2 cm, 75 mm i.d., particle size of 3 μm, and pore size of 100 A; Dionex) and Zorbax column (Zorbax 300SB-C18 stationary phase, length of 15 cm, 75 mm i.d., particle size of 3.5 mm, and pore size of 100 A; Agilent Technologies). Each sample was applied to the precolumn in water for high-pressure liquid chromatography (HPLC) with 0.1% formic acid (v/v) for 5 minutes at a flow rate of 2 μl/min. Then, the precolumn was placed in front of the column. Peptides were eluted with a mixture of solvent A (water for HPLC with 0.1% formic acid (v/v)) and solvent B (79.9% acetonitrile for HPLC (v/v), 20% water for HPLC, and 0.1% formic acid (v/v)), increasing the density gradient of solvent B from 5 to 40% (v/v) for 120 minutes at a flow rate of 300 nl/min. Then, the system was washed for 10 minutes with a mixture of 99% solvent B (v/v) and for 10 minutes with a mixture of 5% solvent B.

The voltage was 2000 V; the temperature of the capillary was 200°C. The mass spectrometer operated in a data*-*dependent acquisition mode: in each cycle, a panoramic spectrum was obtained; the most intense 20 peaks in the panoramic spectrum were selected in turn for fragmentation and recording of product ion spectra and then excluded from the assessment for 10 seconds. A panoramic spectrum was recorded at a resolution of 70,000 in a mass-to-charge ratio value of 400 to 1200 m/z with automatic gain control (AGC) setting of 106 and time limits for filling of 50 ms. Product ion spectra were recorded at the resolution of 17,500 and AGC of 105 with time limits for filling of 100 ms. Collision energy was 30 V; the width of the ion isolation window was 2 m/z.

On the basis of mass chromatograms (.raw file format), a list of centroid spectra in Mascot Generic Format using the MSConvert utility of the ProteoWizard package (version 3.0.7.414, 64 bits) was compiled, which was then interpreted by the Mascot search engine (Matrix Science Inc.). Protein identification was carried out using the CP006916.2 protein sequence database of *M. gallisepticum *S6, which was supplemented by sequences for common protein contaminants. The following parameters of identification were used: tryptic peptides; maximum of one missed cleavage; precursor- ion charge was +2 or +3; the allowable error in the mass of parent ions was 10 ppm; the accuracy of the mass peak measurement of fragments was 0.5 Da; ESITRAP instrument; no permanent modification; cysteine carbamidomethylation and oxidation of methionine residues were variable modifications.

The list of statistically significant identities was defined as a list of proteins with 2 or more identified peptides with p < 0.05.

## RESULTS AND DISCUSSION

**Distribution of RNA in isolated fractions**

**Fig. 1:**
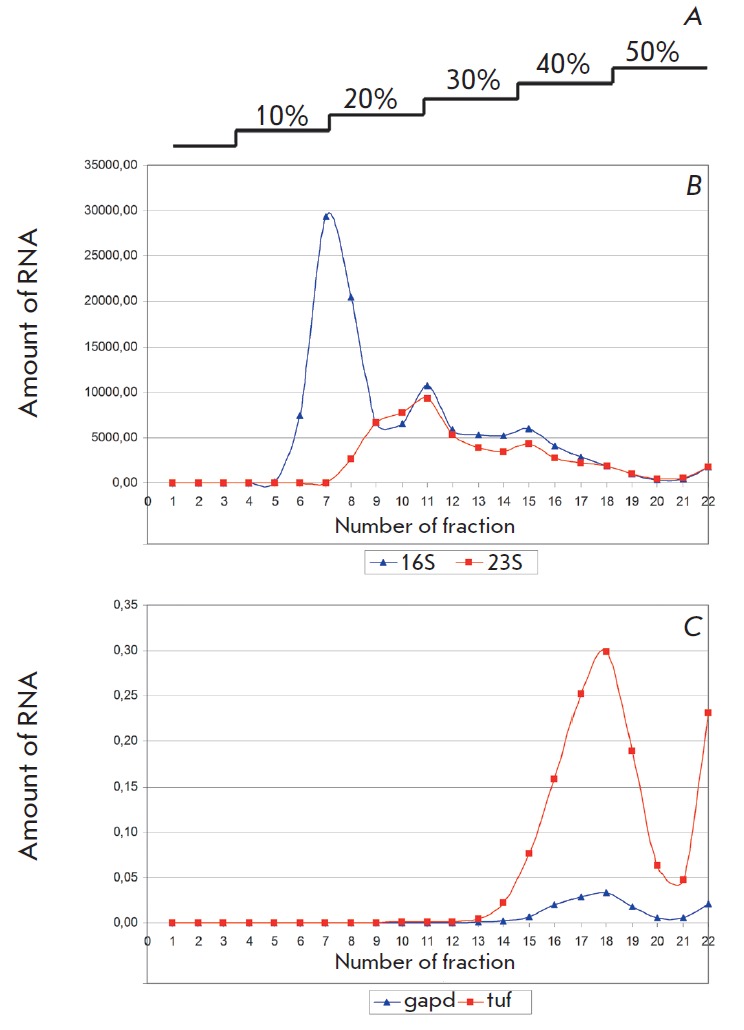
Fractionation of cytoplasm of *M. gallisepticum *in a sucrose gradient. Abundance of 16S rRNA, 23S rRNA,* gapd *mRNA, and *tuf *mRNA was measured. All results (for rRNA and mRNA) were normalized to the 16S mRNA level in fraction 1. A – step gradient of sucrose (regarding obtained fractions); B – abundance of rRNA; C – abundance of mRNA

After the cytoplasm of *M. gallisepticum *was fractionated, distribution of RNA in the fractions was measured by RT-qPCR (see Experimental section). The results are presented in *Fig. 1*. Small ribosomal subunits and large ones cause peaks in fractions 7 and 11, respectively.* M. gallisepticum *is characterized by anapproximately 4-fold abundance of 16S rRNA compared to 23S rRNA [16], which is consistent with the observed picture. Fractions after 12–22 reveal an equimolar ratio of 16S and 23S rRNA. Relatively high levels of mRNA was detected only in polysomes (fractions 15 and above). The highest amount of mRNA was detected in fractions 17 and 18, comprizing about 1 microgram. This makes the corresponding fractions most suitable for further analysis, especially when using high-throughput sequencing technologies.

Suitability of the technique for the quantitative analysis of the abundance of transcripts associated with ribosomes was tested for fraction 18. Transcriptional profiling of 67 genes in three biological replicates using RT-qPCR was conducted. The reproducibility of the data was evaluated using Spearman correlation (see Table). In each pair of samples, the correlation coefficient ranged from 0.87 to 0.92, indicating good reproducibility of the method. The correlation between the abundance of transcripts in the ribosome-bound mRNA fraction and total cytoplasmic mRNA fraction was 0.78.

**Proteomic profiling of *M. gallisepticum *ribosomes**

In order to validate the methods of ribosome purification from *M. gallisepticum*, proteomic profiling of fractions 7 (30S subunit), 17, and 18 (70S ribosomes associated with the mRNA) was conducted and a semiquantitative evaluation of protein abundance according to emPAI was made.

In fraction 7, 18 out of 20 proteins of the small ribosome subunit and only 8 out of 33 proteins of the large subunit were detected, which agrees with the data on the distribution of rRNA in fractions. Thus, fraction 7 was mainly composed of small ribosome subunits. It should be noted that fraction 7 included a significant amount of additional cellular proteins.

Ribosomal proteins were mostly abundant, as expected, in fractions 17–18. A total of 47 out of 53 ribosomal proteins (19 out of 20 proteins of 30S subunit and 28 out of 33 proteins of 50S subunit) were detected. All proteins that were not identified were small in size (less than 100 amino acids); the latter probably impeded identification of these proteins. The abundance of ribosomal proteins in this fraction was regarded as equimolar. Proteins of the small and large subunits had the same emPAI value. Moreover, in fraction 18, a high content of ribosome-associated proteins (EF-Tu and EF-Ts translation factors and Tig and DnaK chaperones) and HU protein was detected. It is known that HU protein, which is a bacterial histone-like protein, can bind both DNA and RNA [17]. It is possible that this protein can bind mRNA or rRNA within the ribosomes.

In fraction 18, we identified a GCW_03230 protein with a high emPAI value. This conservative protein of unknown function is common in many mycoplasmas. Considering the small size (74 amino acids), the feature of this protein is the extreme pI value (11.0), which makes the protein similar to ribosomal proteins. GCW_03230 is likely a new ribosomal protein. In fraction 18, a number of proteins with a relatively high emPAI value, which were not directly related to the process of translation (e.g., triosephosphate isomerase, thioredoxin, a number of proteins of unknown function), were also detected. On the one hand, their presence can be explained by nonspecific interactions with ribosomes after cell lysis. On the other hand, it has been shown recently that such proteins can modulate ribosome activity *in vivo *[11].

**Influence of secondary structures and ribosome binding site on mRNA abundance in the ribosome-bound pool**

Our results indicate that the mRNA abundance in the ribosome-bound fraction generally corresponds to the mRNA abundance in the total RNA fraction. However, some mRNAs were significantly more or less abundant in this fraction. Binding efficiency of mRNA to the ribosome was determined also by complementary interactions between the 3’ end of 16S rRNA and the ribosome binding site in the 5’-untranslated region (5’-UTR) of mRNA and by the presence of secondary structures in this region that mediate or block binding to ribosomes.

Using the RNA duplex program, we modeled *in silico *the interaction between the 3’-terminal region of 16S rRNA (UUA**CCUCCU**UUCU; underlined is the canonical ribosome binding site in *E. coli*) and the 25-nucleotide region upstream of the start codon of each gene. Thus, we obtained results of the binding force of the 16S rRNA with the 5’-UTR of the corresponding mRNA. Spearman correlation between our evaluation of the capacity of the ribosome binding site and the abundance of the corresponding mRNA in the ribosome-bound RNA fraction was 0.39 (*p* < 0.01). We selected mRNA with more than 2-times up-abundance (19) and down-abundance (25) in the ribosome-bound RNA fraction compared to the total mRNA. The energy of duplex formation with the 3’ end of 16S rRNA in 5’-UTR of the up-abundant mRNAs was on average half that of down-abundant mRNAs (dG was –4.96 and –2.52 kcal/mol, respectively).

Despite the expected low efficiency of binding to ribosomes (dG>0), some mRNAs (e.g., *GCW_02495 *and* putA) *were more abundant in the ribosome-bound fraction than in the total RNA fraction. In the case of* GCW_02495*, this paradox can be explained by the fact that the *GCW_02495 *gene is expressed with polycistronic mRNA, together with the adjacent *GCW_02490* gene that has a very effective ribosome binding site (dG=–11.8 kcal/mol). Thus, the corresponding mRNA generally binds well with the ribosome.

**Fig. 2:**
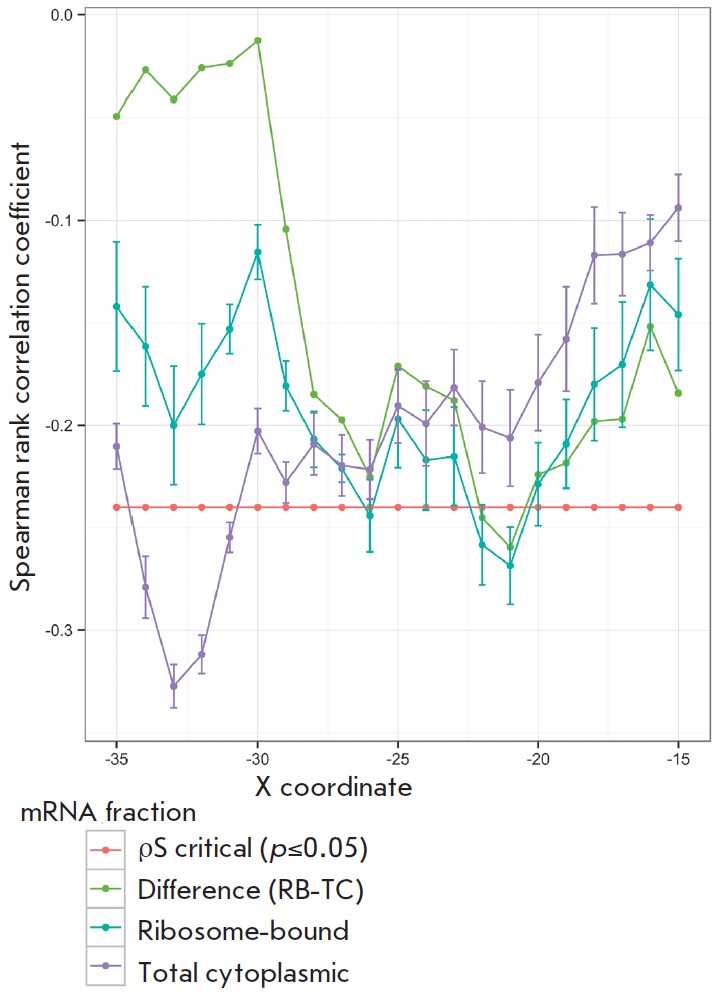
Correlation between mRNA abundance in a given fraction and dG of the secondary structure near the start codon in a sliding window of 30 nt (p < 0.05 for ρ > 0.25)

Several mRNAs were less abundant in the ribosomebound fraction than in the total RNA fraction despite the predicted efficiency of ribosome binding. These mRNAs are *GCW_00085, glpF, gyrA, gyrB, ruvA, potD*, and *hrcA*. This behavior can be explained by the presence of certain secondary structures in the 5’-UTR of mRNA which prevent ribosome binding. Using the quickfold program, we calculated dG values of hairpin structure formation in the region of ribosome binding site and start codon using a sliding window of 30 nucleotides. As a result, we found that the dG value of the secondary structure near the start codon correlates with mRNA abundance in the ribosome-bound fraction (*Fig. 2*). The best correlation was identified in the range of –21 ... +9 nucleotides upstream of the start codon both for mRNA abundance in the ribosome-bound fraction and for the relative mRNA abundance in the ribosome-bound fraction regarding the total RNA level. Thus, the mRNA abundance in the ribosome-bound fraction in *M. gallisepticum *can be modulated by secondary structures in the start codon region.

## CONCLUSIONS

Our results suggest that the amount of ribosome-bound mRNA in *M. gallisepticum *is largely determined by two parameters: (1) the level of gene transcription and (2) the efficacy of the complementary interaction between the 3’-end of 16S rRNA and the ribosome binding site in the 5’-UTR of mRNA. We have developed a quantitative and reproducible method for obtaining the ribosome-bound fraction of mRNA from *M. gallisepticum*, which can be used for studying the process of translation in this bacterium.
